# Discovery of novel alternatively spliced *C. elegans* transcripts by computational analysis of SAGE data

**DOI:** 10.1186/1471-2164-8-447

**Published:** 2007-11-30

**Authors:** Peter Ruzanov, Steven J Jones, Donald L Riddle

**Affiliations:** 1 Michael Smith Laboratories University of British Columbia, Vancouver BC V6T 1Z4, Canada; 2 Genome Sciences Centre, BC Cancer Research Centre, Vancouver, BC V5Z 4S6, Canada

## Abstract

**Background:**

Alternative RNA splicing allows cells to produce multiple protein isoforms from one gene. These isoforms may have specialized functions, and may be tissue- or stage-specific. Our aim was to use computational analysis of SAGE and genomic data to predict alternatively spliced transcripts expressed in *C. elegans*.

**Results:**

We predicted novel alternatively spliced variants and confirmed five of eighteen candidates selected for experimental validation by RT-PCR tests and DNA sequencing.

**Conclusion:**

We show that SAGE data can be efficiently used to discover alternative mRNA isoforms, including those with skipped exons or retained introns. Our results also imply that *C. elegans *may produce a larger number of alternatively spliced transcripts than initially estimated.

## Background

In eukaryotes, alternative splicing creates a diversity of proteins with a limited number of genes. Producing variants of the same protein may be beneficial for tissue specialization at different developmental stages, or when subject to changing physiological conditions. Regulation of alternative splicing also provides an additional layer of control over gene expression. The importance of alternative splicing has been shown in multiple studies of development and cancer [1-3]. Identification of new alternative splice variants may provide additional knowledge about gene regulation and function. Such information is essential for developing treatments for diseases associated with splicing abnormalities, for instance, by using inhibitors of the aberrant transcript expression [4].

Although non-coding sequences (introns) are present in the genomes of all eukaryotes, alternative splicing is more common in complex, multicellular organisms. This bias may be caused by the difficulties in developing such a mechanism by fast-growing unicellular organisms, as production of splice variants, although helpful in achieving protein diversity, also poses a risk of generating aberrant protein products [5].

Splicing studies identified different varieties of transcript rearrangements together with several key proteins involved in this process [6]. The most prevalent form of alternative splicing is exon skipping (cassette exons), comprising about 40% of all splicing events conserved between humans and mice [7]. Comparative studies of exon skipping in mice and humans also indicate the presence of selective pressure for retaining 'functional splice variants', in which exon skipping does not shift the open reading frame (ORF) for the encoded protein [8]. In *C. elegans*, 77% of cassette exon splice variants retain the original ORFs according to the data available in release WS130 of public Wormbase database [9].

*C. elegans *is a well-studied model organism with a fully sequenced genome, and its alternative splicing has been thoroughly investigated using computational analysis of EST (Expressed Sequence Tags) sequences [10]. The results of this analysis are available through Wormbase. Comparison of ESTs with genomic sequence revealed 1782 genes with alternatively spliced transcripts (Wormbase release WS130), which accounts for about 9% of all *C. elegans *genes. By comparison, it is estimated that 40–80% of all human genes may be alternatively spliced [11,12].

We used data from serial analysis of gene expression (SAGE) for computational prediction of novel alternative exon skipping and intron retention events to discover previously unidentified splice variants in *C. elegans*. Unlike microarrays, SAGE provides information for previously unknown polyadenylated mRNA. We analyzed the data from six *C. elegans *SAGE libraries using a set of custom Perl scripts. For computational predictions we used *C. elegans *DNA sequence information from Wormbase release WS130. Applying strict selection criteria, we chose the eighteen most probable predictions of novel alternative splicing events for validation experiments with RT-PCR. Three of the eight predicted exon skipping and two of ten intron retention cases were confirmed in these experiments, demonstrating that computational predictions based on genomic and SAGE data are useful for discovery of novel alternative splice variants. This study is aimed at testing the possibility of predicting alternative splice using computational analysis of SAGE data and genome sequence. To our knowledge, this is the first such study in *C. elegans*.

## Results

### Computational prediction of novel alternative splicing events

**Figure 1:**
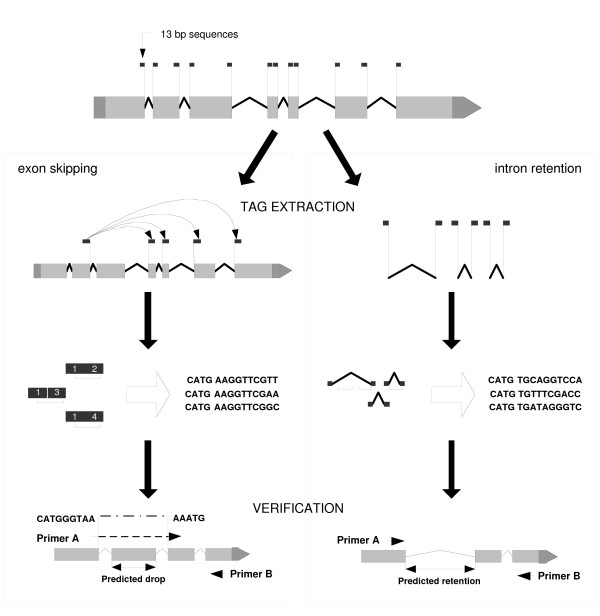
**Schematic illustration of computational prediction and experimental validation of splice variants**. We used virtual splicing events to extract all potential SAGE tags spanning the predicted splice junctions as shown. Introns and flanking 13 bp sequences were used for extraction of SAGE tags identifying cases of intron retention. For exon skipping validation, one of the primers spanned the predicted splice junction, so that hybridization with the template mRNA was possible only if the predicted transcript was present in the mRNA pool. Detection of an RT-PCR product with a larger size than expected for a normally spliced isoform confirmed intron retention events.

We used Wormbase release WS130 as the source of information about the intron/exon structure of *C. elegans *genes and their DNA sequences. Sequences for each of the predicted 22,249 transcripts were composed using gff files downloaded from Wormbase and custom Perl scripts. We generated virtual splicing events for all genes with introns in the database. For the exon skipping simulation, one or more exons were excluded from the final transcript for each gene that had at least 3 exons. The sequences of virtual splicing junctions were then checked for potential SAGE tags (Fig. 1) by scanning 13 bp sequences of each upstream and downstream exon forming the virtual splice junction. The SAGE protocol [13] generated 14 bp tags for transcripts starting with a CATG sequence (NlaIII digestion site), so not every virtual junction is expected to produce a SAGE tag. Nevertheless, it was possible to derive at least one virtual SAGE tag for 6157 *C. elegans *transcripts (28% of *C. elegans *transcriptome).

For the intron retention analysis, we used the introns and their flanking 13 bp sequences for extraction of virtual SAGE tags (Fig. 1), the presence of which in the expression data set would indicate possible intron retention events. In the latter analysis we retrieved 67,709 tag sequences for 14,213 genes (64% of *C. elegans *transcriptome).

We used the virtual transcriptome of *C. elegans *[14] to filter the initial list of predicted SAGE tags. All the tags previously unambiguously mapped to transcripts were removed from the initial list to avoid an overlap. We also analyzed whether the predicted splicing events shifted the ORFs of the analyzed transcripts. Also, we limited our exon skipping candidate list to the variants with undisturbed ORFs, aiming to narrow it to the most interesting functional splice variants. Finally, the SAGE data were examined to determine which tags, corresponding to the predicted splicing events, were actually expressed in six SAGE libraries used for this analysis.

Forty-one unique virtual SAGE tags derived during the analysis of exon skipping were present in at least one of the six SAGE libraries. We chose eight candidate variants for subsequent validation, giving priority to the predicted splice variants with the highest SAGE tag counts and a single dropped exon (Table 1).

**Table 1:** Expression data for exon skipping candidates

**Gene/Tag**	**Junction**	**Exon length**	***F-1***	***F-6***	***DF1***	***DF-6***	***DF10***	***N2-1***
**C33G3.4**								
TGAAAGAAAA	5..7	255	3	0	0	1	1	3
**C52E4.6a**								
ATAAAAATAG	6..8	723	0	0	0	0	1	0
**F27D4.4**								
AGAATGAAAA	4..6	102	3	0	0	0	1	0
**T01G5.1**								
TATTCATTCT	4..6	219	2	3	2	0	1	5
**T05B4.1**								
GGTTTATAAA	3..5	66	0	2	2	0	0	1
**T14G10.1**								
CCAGAAATGG	4..6	264	3	13	6	1	12	22
**W04G5.9**								
GAACTGAATG	10..12	123	3	4	0	1	2	1
**Y49F6B.8**								
CTAAAATGAT	1..3	399	0	1	0	0	0	0

SAGE data for eight tags selected for validation of predicted exon skipping by RT-PCR. Junction column shows the exons that form the predicted splice junction, and the other columns show the normalized tag counts in the SAGE libraries made from the RNA preparations used in validation experiments: F-1, 6: *fer-15 *at days 1 and 6 of adulthood; DF-1, 6, 10: *daf-2; fer-15 *at days 1, 6 and 10 of adulthood; N2-1: N2 at day 1 of adulthood. Tags in all libraries were normalized to 100,000 total tags.

Analysis of the transcripts annotated in the release WS130 of Wormbase with intron retention (Additional file 1) showed that the majority of virtual retained introns had length of 40–60 bp and more than 80% of them have length less than 125 bp. Based on this information, we chose to eliminate tags extracted from introns longer than 125 bp. For each of the remaining 4361 virtual SAGE tags we analyzed its position in the corresponding transcript. Although most of the SAGE tags would be expected to come from the first position (closest to the 3' end), incomplete digestion of the cDNA during library preparation may produce tags positioned further from the 3' end. According to this logic, we chose to keep only virtual SAGE tags with ordinal positions first through third. Forty-one of the 648 tags fulfilling all criteria were present in the SAGE libraries analyzed. We selected ten candidates with the highest tag counts for experimental validation (Table 2). A flowchart illustrating the filtering process is also provided in Additional file 2.

**Table 2:** Expression data for intron retention candidates

**Gene/Tag**	**Intron**	**Intron length**	***F-1***	***F-6***	***DF1***	***DF-6***	***DF10***	***N2-1***	***DAU***
**B0041.3**									
TACGATTTCA	2	48	1	2	1	2	0	1	2
**C08B6.13**									
AGGATACAAT	1	81	4	6	1	1	1	1	0
**C14C6.5**									
CGGTTATTGC	3	69	27	7	31	1	73	10	3
**C24G6.3**									
TGAAATAATA	12	51	0	0	1	5	0	0	2
**D1054.10**									
ATCGGTGTGT	2	91	0	0	4	0	3	0	0
**F07C6.2**									
TAATGAATTT	2	56	1	1	1	2	0	1	2
**R09E10.3**									
TCAATAAATA	8	82	9	0	0	0	0	0	0
**T23G7.5**									
TTTTATATAA	4	47	2	3	3	0	1	8	1
**W01A8.3**									
AAAACAATAA	5	70	8	1	0	0	0	0	0
**Y116A8C.30**									
TATTGGAATC	3	97	0	0	1	0	0	0	3

SAGE data for ten tags selected for validation of intron retention by RT-PCR. The Intron column indicates which intron was predicted to be retained and the Intron Length column shows the length of that intron in nucleotides. The other columns show the normalized tag counts in the SAGE libraries made from the RNA preparations used in validation experiments: F-1, 6: *fer-15 *at days 1 and 6 of adulthood; DF-1, 6, 10: *daf-2; fer-15 *at days 1, 6 and 10 of adulthood; N2-1: N2 at day 1 of adulthood; DAU: young dauer larvae. Tags in all libraries were normalized to 100,000 total tags.

### RT-PCR validation

**Figure 2:**
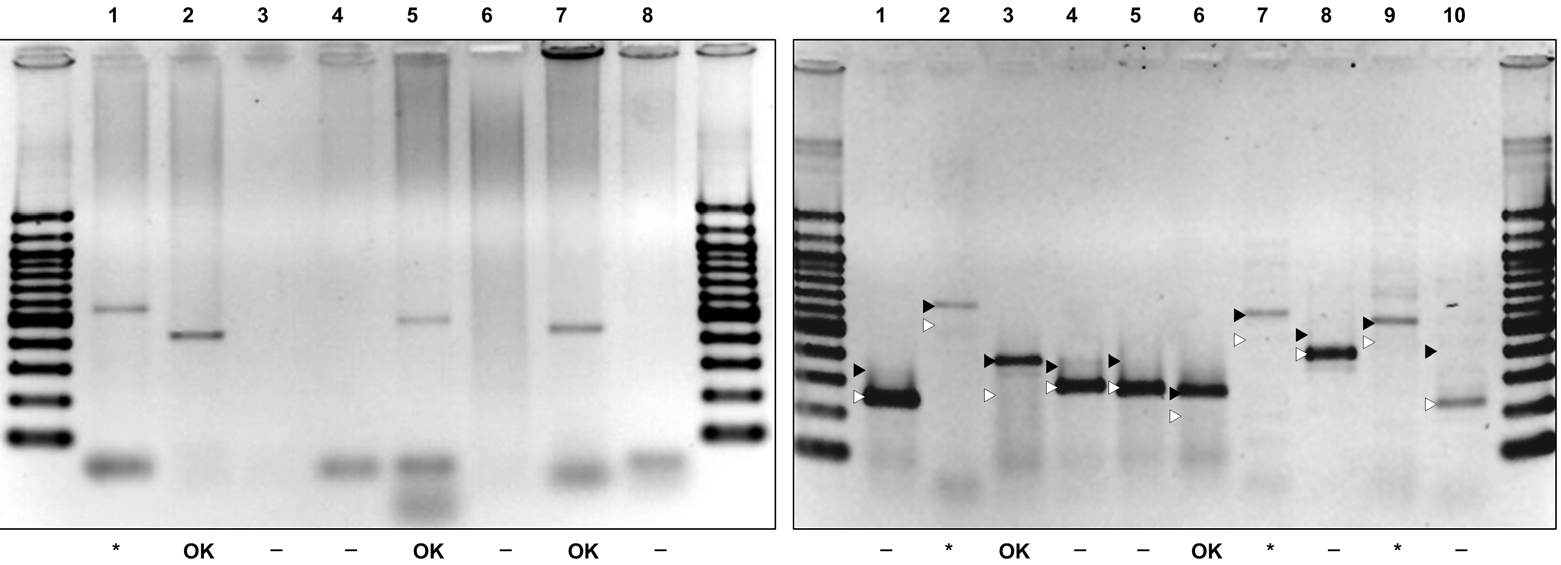
**Validation RT-PCR experiments**. *Left panel*: the results of RT-PCR experiments for eight selected exon skipping candidates are shown. Lanes 1–8 correspond to the genes C33G3.4, C52E4.6a, F27D4.4, T01G5.1, T05B4.1, T14G10.1, W04G5.9 and Y49F6B.8. PCR products of the expected sizes (400 – 600 bp) were observed for four genes. *DNA sequencing did not confirm one of the candidates (C33G3.4, lane 1 on the left panel). *Right panel*: the results of the intron retention analysis. The positions of the PCR product for normally spliced isoforms (empty arrows) and isoforms with intron retention (black arrows) are shown. Lanes 1–10 correspond to the genes B0041.3, C08B6.13, C14C6.5, C24G6.3, D1054.10, F07C6.2, R09E10.3, T23G7.5, W01A8.3 and Y116A8C.30. * Additional RT-PCR experiments with oligo(dT) primer followed by DNA sequencing disproved three candidates (C08B6.13, R09E10.3 and W01A8.3).

We conducted RT-PCR experiments to test our predictions. In the validation experiments for exon skipping candidates, one of the primers overlapped the predicted splice junction (Fig. 1), so a PCR product was expected to appear only if there was a detectable expression of a transcript with the predicted exon rearrangement. In RT-PCR experiments we analyzed the same total RNA samples, which were used for generation of the SAGE libraries. We detected the product of expected size (400 – 600 bp) for the four of eight selected candidates (Fig. 2). All but one of the amplified cDNA fragments had the predicted sequence, confirming the predicted alternative splicing events for C52E4.6a (cyl-1, cyclin L), T05B4.1 (ionic channel protein, also confirmed in a separate RT-PCR experiment with a different primer design) and W04G5.9 (predicted N-glycanase).

In validation experiments for intron retention candidates (Fig. 1), we performed RT-PCR using primers complementary to the flanking exons. The positive candidates were detected by the appearance of a longer PCR product. We obtained positive results for five of ten candidates: C08B6.13 (*srxa-19*, Serpentine Receptor class XA), C14C6.5 (Secreted surface protein), F07C6.2 (predicted protein), R09E10.3 (Long-chain acyl-CoA synthetase) and W01A8.3 (cuticulin). To assess the possibility that our RNA samples contained immature transcripts, we performed additional RT-PCR experiments for each of five pre-selected candidates using one of the gene-specific primers in combination with oligo(dT) primer in at least three trials, expecting a PCR product only from a polyadenylated transcript. We observed a PCR product of the predicted size (confirmed by DNA sequencing) for two candidates – C14C6.5 and F07C6.2. In both cases, the intron retention is corroborated by aligned ESTs (Wormbase web site [9], WS170, February 10, 2007). However, the latter observation may also indicate that Wormbase models for these two genes are incorrect and need to be revised, especially that in both cases there are no EST alignments with exons following the corresponding (predicted as retained) introns. We did not see any PCR product for the other three genes, leaving open the possibility that these three candidates resulted from contamination with immature nuclear polyA(-) RNA.

We analyzed the integrity of the functional domains of the splice variants for both C14C6.5 and F07C6.2. The open reading frame of the C14C6.5 variant is promptly terminated after short intron-encoded peptide sequence GYCK, generating an isoform 14 amino acids shorter than the original 181-amino-acid protein. As we determined by analysis with PROSITE [15], C14C6.5 native protein contains putative Casein kinase II phosphorylation and N-myristoylation sites, which are also both present in the variant retaining the third intron. In the case of F07C6.2, retention of its second intron leads to the loss of 51 amino acids, including a putative phosphorylation site for tyrosine kinase. The open reading frame of this variant stops inside the retained intron after a single valine codon. However, two putative sites for Casein kinase II phosphorylation, an N-myristoylation site and phosphorylation sites for cAMP- and cGMP-dependent protein kinases (PKA and PKB) remain intact in the 115-amino acid F07C6.2 splice variant.

## Discussion

By simulating virtual splicing *in silico *we were able to predict novel alternatively spliced transcripts for previously annotated genes. Hence, this method can produce new information even in the case of a well-studied organism. We analyzed two types of alternative splicing (exon skipping and intron retention) using strict filtering criteria. A similar approach to other types of alternative splicing in *C. elegans *would likely reveal additional splice variants. This approach is also applicable to other organisms, such as the mouse [16], for which adequate gene annotations and SAGE data are available. Although we cannot readily estimate how many additional alternatively spliced variants *C. elegans *may have, we have shown that SAGE data can be efficiently used for discovery of novel transcript isoforms.

SAGE allowed us to examine the transcript levels for a few thousand genes (typically, 4000–7000 per library) in one experiment. This approach may significantly expand our ability to study alternative splicing and improve our understanding of its mechanism. Alternatively spliced transcripts in *C. elegans *have been characterized using EST data [10], indicating that about 10% of *C. elegans *genes are alternatively spliced. As the authors comment, this number may be an underestimate.

It is interesting that both of our confirmed candidates with intron retention also had ESTs aligned with retained introns, supporting the presence of these transcripts in the mRNA population. However, if taken alone the EST data may not provide sufficient evidence of intron retention. In fact, two other candidates, D1054.10 and R09E10.3, although both having ESTs aligned with predicted retained introns, were not confirmed by our RT-PCR tests.

Microarray analyses of exon skipping events have used oligonucleotide probes designed to overlap the annotated or predicted splice junctions [17,18], but their use as a discovery tool is limited because the design of the oligonucleotide probes requires sequence data for the splice variants being tested. The task of analyzing all possible rearrangements for every annotated mRNA is beyond the capacity of all modern arrays, and prediction of all splicing events resulting in a new RNA sequence is nearly impossible.

Recently, Kuo *et al*. [16] used SAGE data to analyze the mouse genome for novel splicing sites in annotated genes. These authors hypothesized that tags neither mapped to known transcripts nor to the genome might span novel splice junctions. They developed an algorithm (SAGE2Splice) for mapping SAGE tags to potential splice junctions. These authors focused on predicting novel splicing sites in the genome rather than discovery of alternative splicing events, which was the goal of our study.

In contrast to microarrays, the SAGE protocol does not require pre-existing information about analyzed transcripts. In principle, experimental data are obtained for every polyadenylated mRNA in the cell. Both the SAGE protocol and the RT-PCR validation experiments sampled the polyA(+) mRNA population. Although the results of our validation experiments showed that confirmed candidates belong to the pool of poly A(+) mRNA, we do not know if those transcripts are functional. Demonstration of their ability to produce active proteins would require additional work, e.g. analysis of polysome-bound fraction of cytoplasmic mRNA. Nevertheless, the computational predictions based on SAGE data can provide the initial guidelines for identification of novel alternative splice variants. Numerous *C. elegans *SAGE data sets are currently available via online public databases such as GEO [19,20]. Mining these data using our approach should improve our understanding of alternative splicing mechanisms.

## Conclusion

Our results demonstrate a practical application of SAGE data analysis for discovery of alternative mRNA isoforms. SAGE allows sampling of the whole mRNA population including uncharacterized transcripts, which would be missed in analysis with alternative large-scale methods such as microarray. Our results also imply that *C. elegans *has a larger number of alternative mRNA isoforms than initially predicted.

## Methods

### Computational resources and data sets

We used *C. elegans *SAGE data generated for various projects at the Michael Smith Genome Science Center, BC Cancer Agency [14,21-24]. We also used the publicly available release WS130 of Wormbase [9]. Data were analyzed using scripts written in Perl 5.6. Tag to gene mapping data were generated using an in-house developed set of scripts as described by McKay, *et al*. (2003). All SAGE libraries were analyzed and filtered for erroneous data (duplicate ditags, single base mismatches etc.) according to standards developed at the Michael Smith Genome Sciences Centre [25]. Sequence reads were processed, and their quality was assessed by use of *Phred *[26,27]. The SAGE data used in this study are available for browsing online via Multisage tool [28].

### RT-PCR

We analyzed the same total RNA samples that were originally used for generation of the analyzed SAGE libraries. These libraries were originally constructed to compare gene expression profiles in long-lived *daf-2 *mutant adults with adults that had a normal life span [21]. We used seven RNA samples: *fer-15 *(*b26*ts) at days 1 and 6 of adulthood, *fer-15; daf-2(m41) *at days 1, 6 and 10 of adulthood, N2 at day 1 of adulthood and two-day-old N2 dauer larvae [22,23,29]. The *fer-15 *(temperature-sensitive sperm deficient) mutation is present in both the *daf-2(+) *and *daf-2(-) *strains to prevent contamination of aging adult populations with progeny.

The following gene-specific primers were used in tests for exon skipping candidates: T14G10.1F: GACTGGAAGGTGTTACAAGA, T14G10.1R: TGTATCTCCATTTCTGGCAT; W04G5.9F: ATGCTGAAGACAACAACTTC, W04G5.9R: CAGCATTCAGTTCCATGATC; T01G5.1F: GTGCTCTTCTTCGAAATGAT, T01G5.1R: TCCACCAGTGTCCTCGAATC; F27D4.4F: GGGACTCGGACAAATTGAAT, F27D4.4R: TCTCATGTTTTCCAGATTTG; C52E4.6aF: TTGTGATAAGTGGTTGATGA, C52E4.6aR: ATTTTTATCATGTTGTTTCGTA; Y49F6B.8F: CATGCTAAAATGATTCCCAA, Y49F6B.8R: CGTATGAATCATAGTTCGAA; T05B4.1F: CATGGGTTTATAAATTCCCA, T05B4.1R: GAAGTGTAAGCACTACACCA; alternative primers for T05B4.1: T05B4.1mF: GAATGGACAGACCAACGCTT; T05B4.1mR: GTCACTTTCCATTCGCCATT; C33G3.4F: TAGATTTGCTCATGTGAAAG C33G3.4R: AGCCACCTTCTTTGCAATCT.

For RT-PCR validation tests of intron retention events, the following primers were used: B0041.3F: ACCACCGTCGTCGTCA, B0041.3R: AACAAGGCGCTGGGAG; C08B6.13F: AGCCAAGAAGCAGGAGAT, C08B6.13R: GATATTGACATATGCCACTCATT; C14C6.5F: GTGACTGCCCAGGAATGA, C14C6.5R: AGTAATGCGGAAAAATTCTGAA; C24G6.3F: GATCCTCAATTGTTCCACCA, C24G6.3R: GTATCGTCCGTTCTGGCA; D1054.10F: AGGGCCAACAATTCCATT, D1054.10R: TACGCAATTGCTGTGTGC; F07C6.2F: TGAGCGGCAATTAAGGAA, F07C6.2R: TCTCCAAACGAAAGCGAA; R09E10.3F: AAAAGCAACTGGCGTCAA, R09E10.3R: CGTTGTCCCAGATCCAGA; T23G7.5F: AAGACGGAATGGGCAGAT, T23G7.5R: TCGAAATTGTGGAATCGG; W01A8.3F: GGAATGTACACCGGCTGA, W01A8.3R: GGAGAAGGAGCAAGGAGC; Y116A8C.30F: GGCGTCACTTCCAGGTC, Y116A8C.31R: TCCGGAGCCCAACAG. We also used a custom oligo(dT) primer with a short adapter GACTCGAGTCGACATCGATTTTTTTTTTTTTTTTT. All PCR products of predicted size were analyzed by DNA sequencing, which confirmed the predicted splicing events.

## Authors' contributions

PR SJJ and DLR developed the main ideas and methodology; PR did the computational analysis and RT-PCR experiments; SJJ and DLR provided feedback and coordination of the project. SJMJ, DLR and PR read and approved the final manuscript.

## Supplementary Material

Additional file 1 Supplementary Figure 1 presented as a PDF file. It shows the statistics for splice variants of exon skipping and introns retention types annotated in Wormbase release 130. Use Adobe Acrobat Reader to open it. Click here for file

Additional file 2 Supplementary Figure 2 presented as a PDF file. It is the diagram of our analysis, which also shows the numbers of SAGE tags remaining after each filtering step. Use Adobe Acrobat Reader to open it. Click here for file
