# Supplementary material for: Discovery of novel alternatively spliced C. elegans transcripts by computational analysis of SAGE data
Source: BMC Genomics. 2007 Nov 30;8:447. doi: 10.1186/1471-2164-8-447 (PMC2216036; doi:10.1186/1471-2164-8-447)
Supplement: Additional file 1 — Supplementary Figure 1 presented as a PDF file. It shows the statistics for splice variants of exon skipping and introns retention types annotated in Wormbase release 130. Use Adobe Acrobat Reader to open it. [file 1471-2164-8-447-S1.pdf]

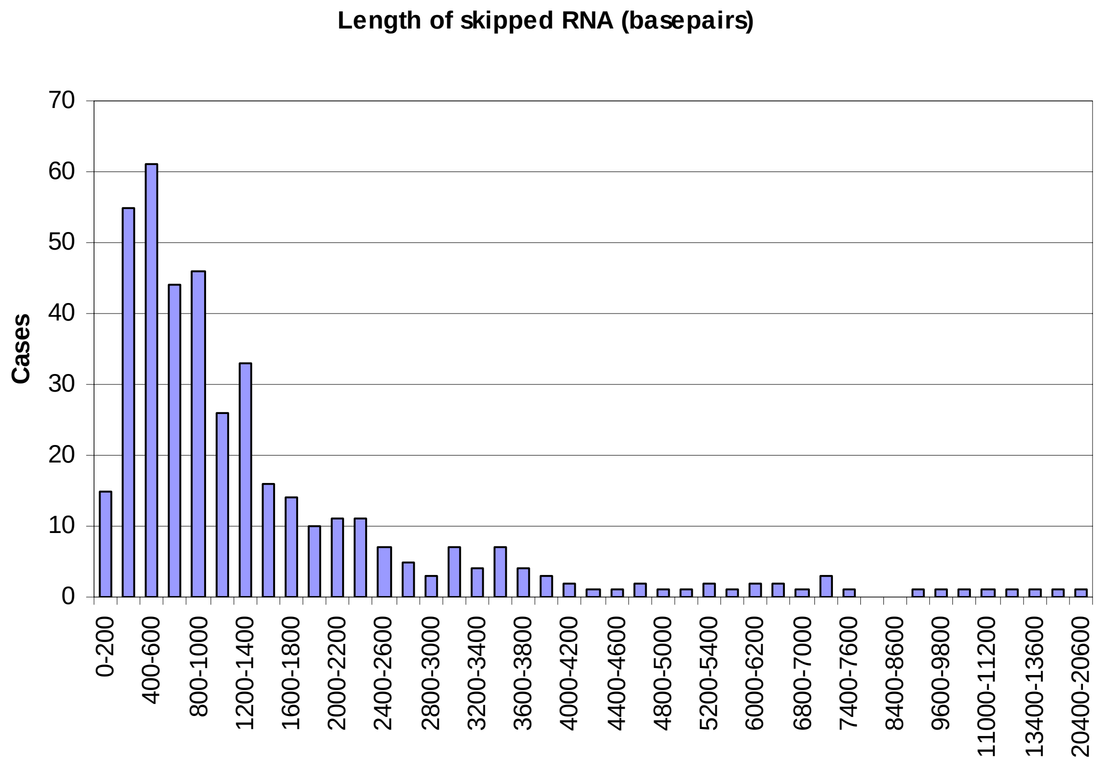

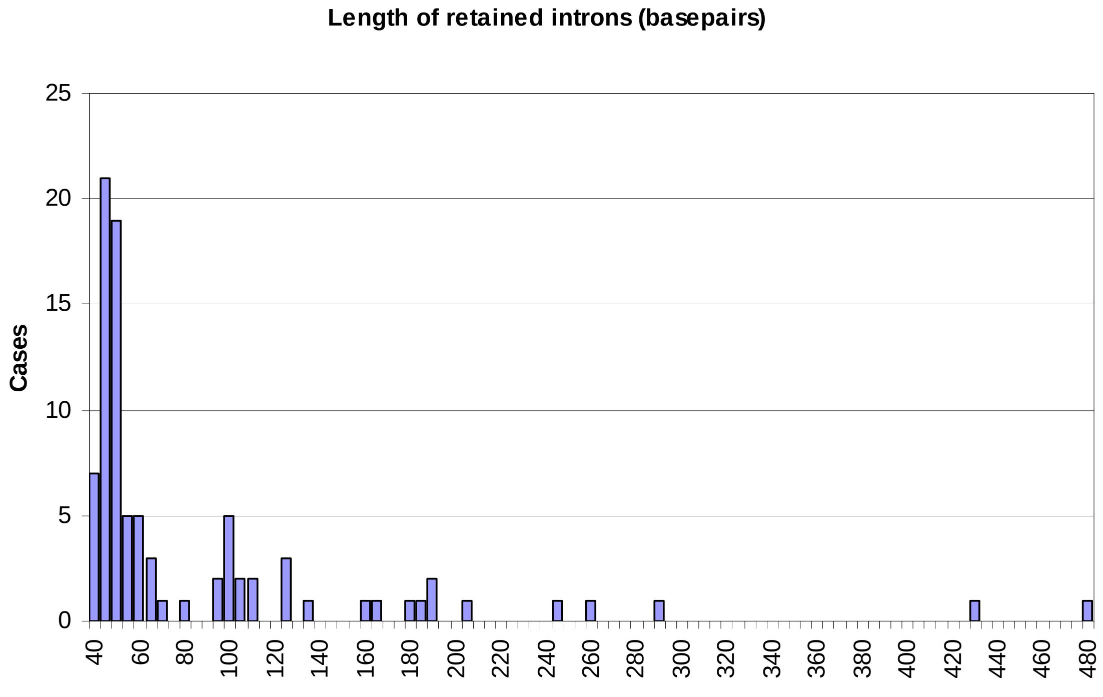

Supplementary Figure 1 Analysis of splice variants annotated in Wormbase release WS130. Top, 410 variants with exon skipping: length of skipped RNA; Bottom, 89 variants with retained introns: length of retained intron RNA
