# Supplementary material for: Discovery of novel alternatively spliced C. elegans transcripts by computational analysis of SAGE data
Source: BMC Genomics. 2007 Nov 30;8:447. doi: 10.1186/1471-2164-8-447 (PMC2216036; doi:10.1186/1471-2164-8-447)
Supplement: Additional file 2 — Supplementary Figure 2 presented as a PDF file. It is the diagram of our analysis, which also shows the numbers of SAGE tags remaining after each filtering step. Use Adobe Acrobat Reader to open it. [file 1471-2164-8-447-S2.pdf]

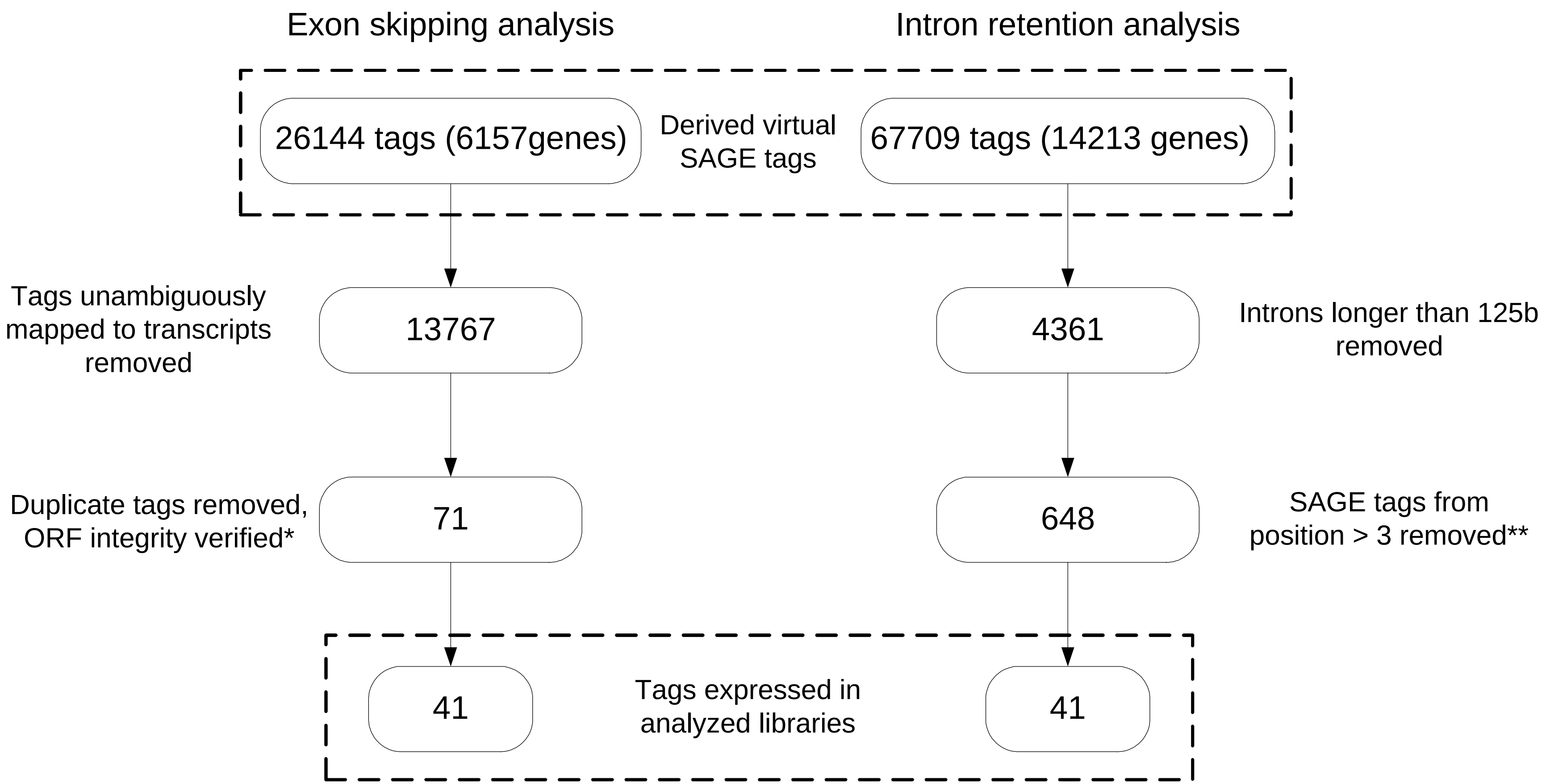

Supplementary Figure 2 Analysis of possible exon skipping and intron retention events for gene models annotated in Wormbase release WS130. The numbers of SAGE tags remaining after each filtering step are shown.

*All candidates with a shift of Open Reading Frame were removed from the list.

**There may be multiple NlaIII sites in a transcript, and due to the partial digestion it is possible to get multiple tags for single cDNA. Tags from positions 1-3 (counting from 3’ end of a transcript) were used in analysis, while tags from more distant positions were discarded.
